# Biosensors for Cancer Biomarkers Based on Mesoporous Silica Nanoparticles

**DOI:** 10.3390/bios14070326

**Published:** 2024-06-30

**Authors:** Minja Mladenović, Stefan Jarić, Mirjana Mundžić, Aleksandra Pavlović, Ivan Bobrinetskiy, Nikola Ž. Knežević

**Affiliations:** BioSense Institute, University of Novi Sad, Dr Zorana Djindjica 1, 21000 Novi Sad, Serbia; minja.mladenovic@biosense.rs (M.M.); sjaric@biosense.rs (S.J.); mirjana.mundzic@biosense.rs (M.M.); aleksandra.pavlovic@biosense.rs (A.P.);

**Keywords:** MSNs, biosensors, cancer biomarkers, electrochemical detection, optical detection

## Abstract

Mesoporous silica nanoparticles (MSNs) exhibit highly beneficial characteristics for devising efficient biosensors for different analytes. Their unique properties, such as capabilities for stable covalent binding to recognition groups (e.g., antibodies or aptamers) and sensing surfaces, open a plethora of opportunities for biosensor construction. In addition, their structured porosity offers capabilities for entrapping signaling molecules (dyes or electroactive species), which could be released efficiently in response to a desired analyte for effective optical or electrochemical detection. This work offers an overview of recent research studies (in the last five years) that contain MSNs in their optical and electrochemical sensing platforms for the detection of cancer biomarkers, classified by cancer type. In addition, this study provides an overview of cancer biomarkers, as well as electrochemical and optical detection methods in general.

## 1. Introduction

Mesoporous silica nanoparticles (MSNs) are a mesostructured porous network of silicon oxide produced through the hydrolytic sol–gel process involving the hydrolysis and condensation of silicon alkoxide precursors under acidic or basic conditions in the presence of a surfactant template [1]. The formation of mesoporous silica typically involves a silica–surfactant micelle templating process, in which condensation of the silicon alkoxide precursor takes place around the surfactant acting as a structure-directing agent [2]. In this manner, hexagonally ordered spherical particles are obtained. MSNs possess precisely controllable physicochemical characteristics, such as particle size, morphology, pore dimensions and volume, surface area, and surface properties [3]. Furthermore, facile surface modification offers the possibility of tailoring the chemical and physical properties of MSNs to achieve specific characteristics or functionalities [4]. The adaptability of MSNs in terms of size, shape, and composition has led to their widespread utilization across various fields [5,6,7,8].

Bioanalytical devices that integrate nanotechnology with biological recognition elements, along with physicochemical transducers to detect and quantify specific biological and chemical substances, are considered nanobiosensors [9]. A transducer is an element that enables the conversion of the target–bioreceptor interaction into a measurable signal. On the other hand, a bioreceptor is a molecule that can interact with a specific analyte and gives the biosensor its specificity [10]. Selectivity, sensitivity, and stability are some of the main characteristics to consider when developing biosensors [11].

Integrating nanomaterials (NMs) in diagnostics opens a potential for increased sensitivity, reduced processing times, and improved cost-effectiveness [12] due to favorable NM characteristics such as increased relative surface area, high surface-to-volume ratio, and quantum confinement effects [13]. Among the variety of NMs, MSNs are of significant relevance in medical diagnostics and sensing applications, presenting a promising tool for the development of advanced nanobiosensors [14,15,16]. However, the successful deployment of MSN-based nanobiosensors depends on a comprehensive understanding of how synthesis methods and post-synthesis modifications influence their performance, considering that the presence of functional groups as well as particle morphology and size is of vital influence on their behavior [13].

On the other hand, cancer remains a leading cause of death worldwide. In 2022, there were an estimated 9.7 million cancer-related deaths globally [17]. The early diagnosis of cancer is crucial for improving patient outcomes. Detecting cancer at an early stage allows for more effective treatment options and potentially better prognosis, increasing the chances of successful therapy and resulting in higher survival rates. Cancer arises from disruptions in normal cell signaling pathways, leading to the emergence of cancer cells with a significant growth advantage [18]. These changes result from a variety of genetic and epigenetic alterations, activating oncogenes and deactivating tumor suppressor genes [19]. However, there is not a single universal gene mutation found across all cancers, and patterns of genetic changes vary not only by tumor location but also within tumors from the same location. With over 200 different cancer types affecting various parts of the body, clinical testing becomes intricate. Given the complexity of, and variability in, cancer-related changes, selecting specific biomarkers for diagnosis is challenging [20]. The National Cancer Institute defines a biomarker as “a biological molecule found in blood, other body fluids, or tissues that is a sign of a normal or abnormal process, or of a condition or disease”. They are produced as the body’s immune response or by the cancerous cells themselves [21]. Determination of cancer biomarker levels in biofluids can be used to detect cancer at different stages or to monitor the outcome of therapy [22]. An important step in ensuring good sensitivity and selectivity for early-stage cancer detection is the identification of appropriate biomarkers as well as the type of biofluid [23]. In addition, the recognition interaction between the biorecognition molecule and the biomarker may also dictate sensitivity [24]. Furthermore, during disease diagnosis, a range of biomarkers is typically analyzed. This means that reliable non-invasive cancer diagnosis often requires simultaneous determination of multiple biomarkers found in different body fluids using different techniques, which are still looking for standardization and validation [25], although there are some techniques that are used in clinical diagnosis as a gold standard. For example, protein biomarkers are principally quantified by the enzyme-linked immunosorbent assay (ELISA), where the target molecule is specifically bound to its natural counterpart—antibody—while the secondary antibody labeled with a certain enzyme acts as a messenger providing a colorimetric signal when the appropriate substrate is added to the reaction well [26]. While the ELISA method can be highly sensitive and selective in complex matrices, it is limited by the moderate risk of false-positive signal production due to its colorimetric nature of detection or nonspecific binding to the reaction well and the high cost of production. To detect DNA/RNA biomarkers, the amplification of nucleic acid is performed to increase the probability of signal detection. Among different methods, the polymerase chain reaction (PCR) method stood out as a golden standard in nucleic acid amplification tools, where using the specific set of oligonucleotides (primers) and enzyme polymerases, the amplification of the DNA segment previously denatured through multiple temperature variation cycles is achieved [27]. However, PCR methods often require a specific laboratory environment and equipment since it is very sensitive to contamination. Despite challenges originating from these and other standard methods, their application is still the first choice due to the lack of appropriate options in clinical use. Additionally, there is a demand for the (RE)ASSURED ((Real-time connectivity, Ease of specimen collection, and environmental friendliness), Affordable, Sensitive, Specific, User-friendly, Rapid and robust, Equipment-free, and Deliverable to end-users) criteria proposed by the World Health Organization to describe and develop an ideal point-of-care testing (POCT) system, primarily in medical applications [28].

There are different strategies in which MSNs are incorporated into biosensors to close the ASSURED circuit. Based on the transduction signal, the most common biosensors for cancer biomarker detection are electrochemical, optical, and colorimetric [29].

## 2. Cancer Biomarkers

### 2.1. General Discussion on Cancer Biomarkers and Relevant Biosensors

Current clinical practice in oncology emphasizes the importance of early diagnosis, proper prognostication, and screening for malignancy in its pre-invasive stage (before metastasis). The important role of biomarkers is widely recognized in research, medicine, and pharmacology. Apart from replacing clinical endpoints and reducing the time and the costs for Phase I and Phase II clinical trials, their levels are measured to diagnose a disease or to monitor treatment efficacy and disease progression [30].

Markers usually differentiate an affected patient from a healthy person. Upon tumor formation, levels of tumor markers rise accordingly, stressing the importance of limits of detection (LOD) for early screening stages. In the case of carcinoembryonic antigen (CEA), the threshold is as low as 3 µg/L of the sample or it can go up to 12.5 mg/L in the case of neuron-specific enolase (NSE) [31]. Apart from sensitivity, specificity is another important perspective. Tumor markers can be associated with different tumors, and most of the tumors have more than one marker associated with their onset and growth. The specificity and sensitivity of a lot of markers are being evaluated for clinical use [32]. Unfortunately, none of the currently described biomarkers achieve 100% sensitivity or specificity. For example, the sensitivity of the prostate-specific antigen (PSA), a serum biomarker for prostate cancer, is greater than 90%, but it has a specificity of only around 25%, resulting in patients needing to undergo a biopsy for the final confirmation of disease [33].

Cancer biomarkers can be different types of molecules such as proteins, DNA, RNA, micro-RNA, peptides, hormones, oncofetal antigens, cytokeratins, exosomes, and carbohydrates. One of the first discovered tumor antigens was carcinoembryonic antigen CEA, a glycoprotein molecule isolated in 1965 [34]. Biomarkers can be intra or extracellular. In cases in which biomarkers are intracellular, cells need to be lysed to collect them. Biomarkers are detectable in tissues and/or biological fluids like blood (whole blood, serum, or plasma) and secretions (stool, urine, sputum, or nipple discharge), and thus can be collected non-invasively [35].

There are a few traditional cancer screening methods such as mammography and the fecal occult blood test followed by colonoscopy. Nowadays, scientists are creating tools at the molecular level to measure molecular alterations in the process of tumor growth. Genetic alterations can be inherited, confirmed as sequence variations in isolated DNA, or somatic, identified as mutations in isolated DNA [36]. Although DNA methylation can be studied by Southern blotting, DNA sequencing, DNA microarrays, and PCR, these genomic methods are complex and time-consuming, and genetic markers do not give information on post-translational modifications on proteins. Therefore, protein-based biomarkers are often referred to as the “classic” ones in the literature.

Some of the common biomarkers for different cancer types are listed in Table 1.

As can be seen from Table 1, CEA and CA 19-9 are common cancer biomarkers for several different tumors. Because of that, better than relying on one single biomarker, a panel of biomarkers is more promising as disease predictors [52].

In this review, nanobiosensors that involve MSNs for the detection of biomarkers that are useful in the diagnosis of cancer are discussed.

### 2.2. Specific Cancer Biomarkers Targeted by MSN-Based Biosensors

Recent research studies (in the last five years) involving MSN-based biosensors have been focused on several cancer biomarkers, as detailed in this section and further elaborated with MSN-based biosensor assemblies in the following sections.

Glutathione is a tripeptide (consisting of glutamate, cysteine, and glycine) containing a sulfhydryl group by means of which it is conjugated to other molecules [53]. It is distributed in most mammalian cells and is present in intracellular concentrations from 0.1 to 10 mM [54]. It is an important non-enzymatic antioxidant with a central role in the regulation of reactive oxygen species (ROS) [55]. GSH has been found in increased levels of breast, ovarian, head and neck, and lung cancers [56].

Cytokeratins are polypeptides expressed by all epithelial cells [57]. There are 20 cytokeratins, distinguished by their molecular weight and isoelectric points, which are classified into two groups: acidic and basic–neutral [58]. The fragment of cytokeratin subunit 19, known as CYFRA 21-1, has been recognized as an accurate and specific tumor marker for detecting non-small-cell lung cancer (NSCLC), particularly the squamous cell subtype [59]. CYFRA 21-1 is frequently investigated as a lung cancer biomarker utilizing integrated sensing platforms based on MSNs.

CA 15-3, also known as MUC1, is the most used serum marker for breast cancer. It is a large transmembrane glycoprotein that is often overexpressed and abnormally glycosylated in cancerous cells. Under normal conditions, it is involved in cell adhesion, but its elevated levels in cancer may contribute to metastasis [60].

The human epidermal growth factor receptor 2 (HER2) is part of the epidermal growth factor (EGF) family. HER2 positivity is observed in approximately 15–20% of breast cancers, characterized by the overexpression of the HER2 protein. The HER2 protein promotes cell growth. However, when HER2 is overexpressed, it can lead to aggressive growth of cancer cells [61].

The epithelial cell adhesion molecule (EpCAM) is a type I transmembrane protein consisting of 314 amino acids that are involved in cell signaling and carcinogenesis [62]. EpCAM is notably expressed in most human epithelial cancers, including colorectal, breast, gastric, prostate, ovarian, and lung cancers [63].

Total serum acid phosphatase was the first clinically useful prostate tumor marker to be discovered [64]. ACP is a lysosomal enzyme that breaks down organic phosphates in an acidic environment [65]. ACP is found to be present in the prostate in 100 times higher quantities than in any other tissue type [66]. PSA, or prostate-specific antigen, is a serine protease primarily produced by prostate cells and released into the ejaculate to help liquefy semen [67]. Normally, PSA levels in the blood are low. However, changes in the normal structure of the prostate, such as those caused by cancer, can result in increased levels of PSA in the blood [68].

The presence of high-risk human papillomavirus (HPV) is the most important cervical biomarker as it is strongly associated with the development of this type of cancer [69]. One of the high-risk types, HPV16, is closely associated with invasive cervical cancer. It is well known that the expression of HPV16 E6 oncoprotein is essential for transforming normal cells into cancerous ones [70]. Moreover, it has been proved that the HPV E6 oncogene induces the functional suppression of the tumor suppressor gene p53 [71].

Kato and Tarigoe first identified the squamous cell carcinoma (SCC) antigen in 1997 [72], which was found to be present in both neutral and acidic sub-fractions of tumor antigen 4 (TA-4). Squamous cell carcinomas account for 85–90% of all cervical cancers, while elevated serum levels of SCC have been observed in 28–88% of cases of cervical squamous cell carcinoma [73].

CA 19-9 is the most common diagnostic biomarker for pancreatic cancer, which is used for prognosis and prediction of treatment outcomes. Among other carbohydrate antigens, CEA is also being extensively investigated as a pancreatic tumor marker, but it has not been found to be more sensitive than CA 19-9 [74].

Glypican 1 (GPC1) is a membrane-anchoring protein, which is highly expressed in pancreatic cancer tissue compared to normal tissue [75].

Current strategies for screening ovarian cancer involve using a combination of blood biomarkers, CA 125, and HE-4, along with transvaginal ultrasound imaging. CA 125, also known as MUC16, has been found in up to 80% of women diagnosed with late-stage epithelial ovarian cancer at elevated levels. HE4, also known as WAP 4-disulfide core domain 2, is linked to cancer cell adhesion, migration, and tumor growth [76].

## 3. Detection Methods

### 3.1. Electrochemical Detection Methods

Concerning electrochemical biosensors, the transduction element is often denoted as an electrochemical cell predominantly consisting of three electrodes—working (WE), reference (RE), and counter electrode (CE)—fabricated on a substrate in a co-planar configuration [77]. Various materials are used for the electrode production, from metallic (gold, platinum, etc.), metal-oxides (TiO_2_, ZnO, etc.), carbon-based (glassy carbon, carbon nanomaterials), to polymeric, and different microfabrication methods such as screen-printing, ink-jet printing, photolithography, and others are deployed [78]. In the electrochemical approach, a signal is obtained as a result of electron transfer between the working electrode (transducer) and electrolyte (sample) and it can be measured as current, potential, or impedance of the electrochemical cell. Direct or indirect transduction can be applied, the former is associated with enzymatic biosensors and the latter with mediator-based biosensors [79]. Mediators are small molecules with a low molecular weight, which transfer electrons from the reaction site to the electrode [80] and are usually referred to as redox probes. With the advancement of nanotechnology, in-house or commercial electrode systems have been modified with distinct nanomaterials to improve the overall electrochemical biosensor performance [81]; these systems rely on nanomaterial-enabled signal amplification by exploiting the improved electrochemical properties [82]. Today, many electrochemical techniques are used to study the biosensing mechanism at the surface of the WE and they can be grouped into potentiometric, amperometric, voltammetric, and impedimetric techniques [83]. In potentiometric measurements, the potential between two electrodes, usually WE and RE, is recorded and it can provide definite information about the target analyte presence. The working principle of amperometric biosensors relies on the current amplitude resulting from the redox processes of electroactive species on the WE at the applied potential. Voltammetric techniques are widely used in electrochemical biosensors since the current between WE and CE is monitored during a predetermined potential sweep applied between WE and RE. Depending on the potential type, there are several methods used in biosensing: cyclic voltammetry (CV), differential pulse voltammetry (DPV), square wave voltammetry (SWV), or anodic/cathodic stripping voltammetry. In impedimetric measurements, the total impedance of the electrode/electrolyte electrical circuit is monitored at applied voltage. In Table 2, an overview of the electrochemical techniques applied to MSN-based biosensing of cancer biomarkers is given.

### 3.2. Optical Detection Methods

Optical biosensors use optical properties of the transducer and the optical signal is detected, e.g., electromagnetic radiation in the optical range. From the perspective of signal origin, optical biosensors are usually divided into two groups: label-based and label-free biosensors [102]. Labeled optical biosensors use specific molecules (labels) responsible for signal generation; these can be colorimetric or fluorescent biosensors [103,104]. On the contrary, label-free optical biosensors utilize the change in optical radiation properties of the transducer upon biochemical interaction, such as amplitude, frequency, phase, and polarization [105], but can also be manifested by a measurable physical property of the biosensing interface, such as refractive index.

Colorimetric biosensors are an attractive sub-field because they provide a simple setup and fast analysis, but since the signal is based on a visible color change, such an approach may be insufficient for analyte quantification, complicating the colorimetric scheme of detection. Nevertheless, colorimetric biosensors offer the most criteria for the development of POCT devices precisely because of the visible signal that is easily read by the end user. The quantification of colorimetric data is usually performed using a spectrometric technique to determine the signal, such as UV-Vis absorption spectra [106]. Principally, colorimetric biosensors can be denoted as both label and label-free devices. Owing to the nanoparticle’s unique properties, which can be expressed in the optical signal, their role in colorimetric biosensors is of a transducing nature, producing an optical signal detected by the above-mentioned spectroscopy. Based on the mechanism of color change, colorimetric biosensors can be divided into metal nanoparticle aggregation, enzyme catalytic activity, and chromatic transitions of conjugated polymers [107].

Luminescence is the ability of a material to emit light upon absorption of energy coming from different sources. In biosensor technology, the most significant luminescent phenomena used are fluorescence, chemiluminescence, and electrochemiluminescence. A fluorescent signal is produced by a fluorophore tag or dye anchored to the biological element and may be regulated by a quencher in the so-called “turn-on/turn-off” mechanism. There are different physical principles enabling this strategy for fluorescent-based biosensing, such as Förster resonance energy transfer (FRET) [107], fluorescence inner filter effect (IFE) [108], and others. Upon a chemical reaction, certain materials emit light, which is called chemiluminescence (CL). Furthermore, if an electric field is applied to induce electron transfer between luminescent material and electrochemical probes, the principle is called electrochemiluminescence (ECL) [109].

Considering the application of label-free biosensors for cancer biomarker detection, the most used optical principles are surface plasmon resonance (SPR) and surface-enhanced Raman spectroscopy (SERS) [110]. In SPR, a unique mode of electromagnetic field, surface plasmons (SPs), are excited by the external light in the thin metal film and propagate at the metal–dielectric interface. Due to the biosensing binding event, a change in the refractive index of the medium causes a change in SP velocity, which is measured as a change in external light properties [111]. The SERS principle is based on a Raman scattering enhancement coming from multiple physical principles, like surface plasmon resonance, substrate–molecule charge-transfer resonance at the Fermi energy level, and allowed molecular resonance. Although based on spectroscopic signals [112], SERS biosensors can also be label-based using molecules with distinct Raman signals.

The role of MSNs in optical biosensing is mostly connected to label loading for its controlled release or specific molecular/nanomaterial-based signal enhancers, which is why they are not often used in label-free optical biosensors. In Table 3, an overview of the optical techniques applied to MSN-based biosensing of cancer biomarkers is given.

## 4. MSN-Based Biosensors by Cancer Type

### 4.1. Lung Cancer

In 2022, lung cancer was the most diagnosed cancer, representing 12.4% of all cancers worldwide [17]. In 2020, the highest incidence subtypes of lung cancer were adenocarcinoma (39%), squamous cell carcinoma (25%), small-cell carcinoma (11%), and large-cell carcinoma (8%) [129].

A core–shell ultrasensitive nanozyme (CPT/DM-FA) was developed for fluorescence, UV−vis, and color brightness triple-mode GSH sensing and specific cancer cell detection [114]. The nanozyme consisted of a dendritic mesoporous silica nanoparticle (DMSN) core serving as a camptothecin carrier and a platform for synthesizing a MnO_2_ shell. Integration of FRET and oxidase-mimic-mediated ^1^O_2_, O_2_^−^ generation facilitated fluorescence, UV-vis, and colorimetric GSH sensing with a linear range from 2 to 250 μM and a limit of detection of 0.654 μM. The surface folic acid modification enabled specific cancer cell detection. The platform exhibited switch-on signal response and high sensitivity, suitable for real serum samples (A549 cells (lung cancer) and PC-12 cells). Challenges include nonspecific response due to similar sulfhydryl groups in cysteine and homocysteine.

**Figure 1:**
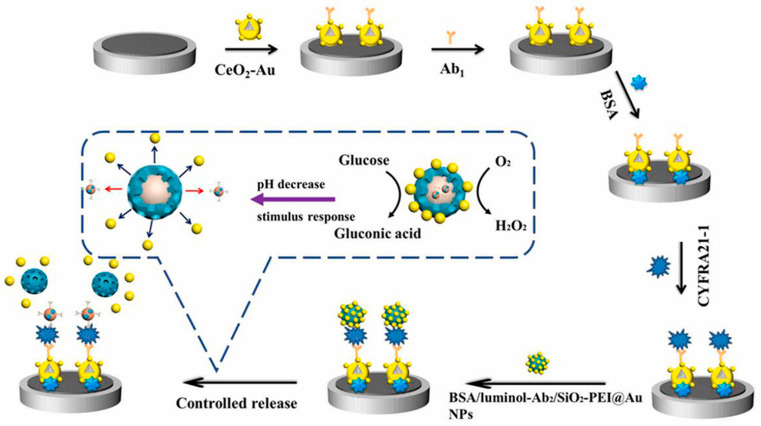
Schematic representation of glucose oxidation-induced pH-responsive MSN-based ECL biosensor for detection of CYFRA 21-1. Reproduced with permission [125].

A self-on ECL biosensor was developed for the efficient detection of CYFRA 21-1 [125]. The biosensor utilized a pH stimulus response-controlled release strategy, employing polyethylenimine-modified silica (SiO_2_-PEI) as a carrier, BSA/luminol-Ab2 as the encapsulated substance, and AuNPs as the blocking agent (Figure 1). Glucose served as the inducer for controlled release. The glucose oxidation led to the production of gluconic acid, triggering a decrease in pH, which caused the release of BSA/luminol-Ab2 from SiO_2_-PEI due to the detachment of AuNPs. The specific binding between CYFRA 21-1 antibody and antigen facilitated ECL signal generation. The biosensor demonstrated detection capabilities within a range of 0.001–100,000 ng/L and a limit of detection of 0.4 fg/mL.

**Figure 2:**
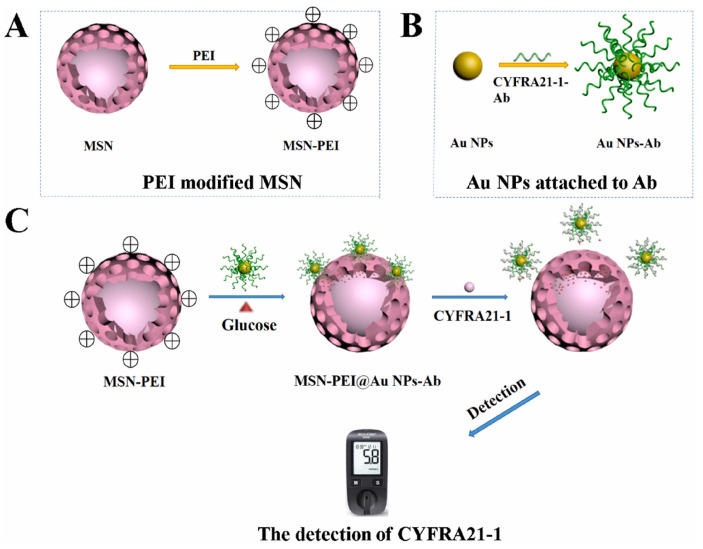
Schematic representation of a controlled-release MSN-based immunosensor for the rapid detection of CYFRA21–1 with the help of a personal glucose meter. Reproduced with permission [85].

Another electrochemical immunosensor for detecting CYFRA 21-1 was developed by Yola et al. [95]. The sensor utilized a silicon nitride (Si_3_N_4_)–molybdenum disulfide (MoS_2_) composite on multi-walled carbon nanotubes (MWCNTs) as a sensor platform, along with core–shell-type magnetic mesoporous silica nanoparticles@gold nanoparticles (MMSNs@AuNPs) as a signal amplifier. The process involved immobilizing capture antibodies on the sensor platform via stable electrostatic/ionic interactions, followed by specific antibody–antigen interactions with the signal amplifier to form a sandwich-type voltammetric immunosensor. The immunosensor exhibited a linear detection range of 0.01–1.0 pg/mL and a detection limit of 2.00 fg/mL. The sensor demonstrated selectivity and sensitivity in plasma samples, highlighting its potential for early detection of lung cancer. An immunosensor based on a personal glucose meter (PGM) was also designed for the detection of CYFRA 21-1 [85]. Glucose was entrapped into polyethyleneimine-modified mesoporous silica nanoparticles (MSN-PEI) using CYFRA 21–1 antibody-labeled gold nanoparticles (AuNPs-Ab) (Figure 2). In the presence of the CYFRA 21-1 antigen, AuNPs-Ab leaves the surface, caused by recognition and binding processes between the antibody and the antigen. Consequently, glucose molecules were released from the pores of MSNs, which are measured by PGM. The proposed immunosensing system exhibited a linear response to CYFRA 21-1 ranging from 1.3 ng/mL to 160 ng/mL with a detection limit of 0.79 ng/mL.

### 4.2. Breast Cancer

In 2022, breast cancer was the fourth most diagnosed cancer, representing 7.3% of all cancers worldwide. Additionally, breast cancer is the most frequently diagnosed cancer and the primary cause of cancer-related death among women [17].

An electrochemically synthesized vertically oriented silica-based mesoporous material (SBMM) modified electrode, combined with a chitosan–luminol (CS-Lu) composite, was utilized for the cytosensing of breast cancer cells [37]. An ECL cyto-immunosensing method was developed for the detection of metastatic breast cancer cells, specifically SKBR-3 cells. The method utilizes a silica-based mesoporous nanostructure synthesized via an environmentally friendly in situ electrosynthesis approach, offering high loading capacity and mechanical strength. Luminol, combined with chitosan, forms a stable lumino composite film on the electrode surface, enhancing stability and sensitivity. Chitosan serves as an adhesive, enhancing stability and sensitivity, while also facilitating the covalent attachment of antibodies for specific cell detection. The protocol demonstrated a lower limit of quantitation of 20 cells/mL and a linear dynamic range of 20 to 2000 cells/mL. Specificity was confirmed against other breast cancer cell lines (MCF-7 and MDA-MB-231), while repeatability was shown with a relative standard deviation of about 1.6% for 500 cells/mL.

**Figure 3:**
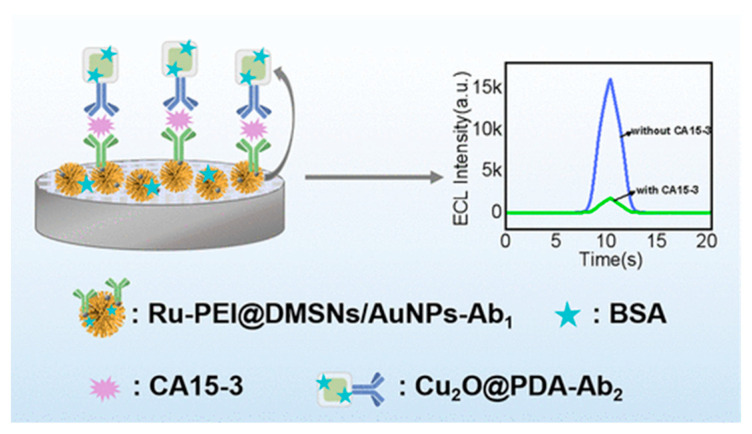
Schematic representation of a dual-quenching ECL immunosensor for the detection of CA15-3 based on dendritic mesoporous silica nanoparticles. Reproduced with permission [124].

Another ECL immunosensor was developed for the specific detection of CA 15-3, a biomarker associated with breast cancer (Figure 3) [124]. The sensor utilized a dual-quenching strategy, incorporating Ru(dcbpy)_3_^2+^, PEI, and AuNPs immobilized on DMSNs to enhance ECL efficiency. The high loading amounts of Ru(dcbpy)_3_^2+^, conductivity, and localized surface plasmon resonance (LSPR) effect of AuNPs contributed to improved ECL intensity. Specifically, a sandwich structural sensing platform (Ab1-CA 15-3-Ab2) was formed, where CA 15-3 served as the target antigen. Cu_2_O nanoparticles coated with poly(dopamine) (Cu_2_O@PDA) were introduced to the sensor through antigen–antibody interaction, leading to significant ECL quenching due to the dual quenchers of Cu_2_O and PDA. The sensor exhibited sensitivity with a linear detection range from 5.0 × 10^–5^ to 6.0 ± 102 U/mL and a limit of detection of 2.4 × 10^–6^ U/mL. Moreover, the sensor demonstrated good selectivity and stability for CA 15-3 detection in serum samples, indicating its potential for clinical applications in the diagnosis and monitoring of breast cancer biomarkers.

Li et al. prepared a peroxidase-mimicking mesoporous silica–gold nanocluster hybrid platform (MSN–AuNC–anti-HER2) modified with recognizable biomolecules for colorimetric detection of HER2-positive (HER2+) breast cancer cells [113]. They immobilized anti-HER2 antibodies onto the surface of MSNs while loading gold nanoclusters inside the pores of MSNs. The prepared MSN–AuNC–anti-HER2 platform was able to catalyze H_2_O_2_ reduction and oxidation of the peroxidase substrate, colorimetric agent, 3,3′,5,5′-tetramethylbenzidine (TMB). It has been suggested that MSN enzyme immobilization and enrichment are crucial for achieving low detection limits. Additionally, it has been demonstrated that the designed system has a high affinity to HER2 receptors.

In another study, mesoporous silica-based ECL was utilized for MCF-7 breast cancer cell detection. MSNs were modified with phenyl-boronic acid, loaded with ECL-active molecules (ruthenium-based dye, Ru(dcbpy)_3_^2+^), and capped by polyhydroxy-functionalized AuNPs [123]. In the presence of ascorbic acid, MCF-7 cells endogenously produce a large number of H_2_O_2_, which subsequently induces the oxidation of arylboronic ester linker causing the release of Ru(phen)_3_^2+^ and increasing the ECL signal. The system exhibited a detection limit of 208 cells/mL for MCF-7 breast cancer cells. A more recent study introduced MONA (Mesoporous silica with Optical Au Nanocrescent Antenna), an integrated nanostructure designed for multifunctional cellular targeting, drug delivery, and molecular imaging. MONA combines an asymmetric Au nanocrescent (AuNC) antenna with a mesoporous silica nanosphere [127].

The MSN serves as a molecular carrier with a large pore volume, facilitating efficient drug delivery, while the AuNC functions as a nanosensor and optical switch. Key findings include specific targeting of EpCAM in MCF-7 breast cancer cells, achieved through conjugation of anti-EpCAM onto MONA, rapid apoptosis of MCF-7 cells facilitated by light-driven molecular, doxorubicin (DOX) delivery, utilizing a highly focused photothermal gradient generated by the asymmetric AuNC, and monitoring of apoptotic events, particularly cytochrome c activity in response to DOX releases by measuring plasmonic energy resonance transfer (PRET) between the AuNC and cytochrome c molecules. A novel strategy to enable EL on MSNs is based on the encapsulation of aggregation-induced EL molecules TPE and TEA as a co-reactant, developing an MSN-TPE-TPA self-enhanced EL system [126]. Furthermore, the detection of MCF-7 cells is realized through strategic capture of CD44 transmembrane glycoprotein via novel WC-7 heptapeptide additionally functionalized with double-stranded DNA probes, of which one is modified with ferrocene, an EL quencher, and acts as a signal initiator. In the presence of target cells, a complex peptide-dsDNA binds to CD44 protein, and by the strand displacement strategy, an Fc-carrying DNA probe is released and extracted making space for its hybridization to a capture probe on the MSN-based EL system.

### 4.3. Prostate Cancer

In 2022, prostate cancer was the fourth most diagnosed cancer, representing 7.3% of all cancers worldwide. Further, prostate cancer was the second most common cancer globally and the fifth leading cause of cancer-related deaths among men [17].

**Figure 4:**
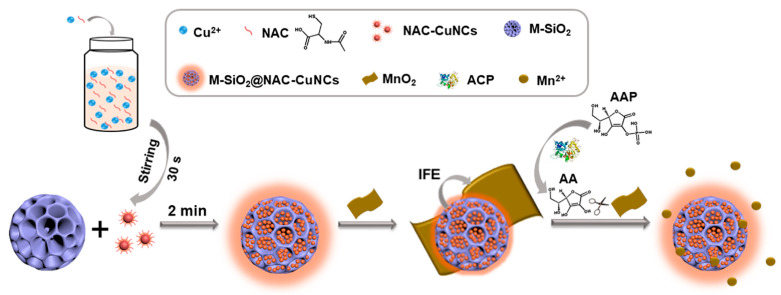
Schematic representation of construction and working principle of a fluorescent nanoprobe based on CuNC-incorporated 3D MSNs. Reproduced with permission [115].

Fluorescent nanoprobes composed of N-acetyl-l-cysteine capped-copper nanoclusters (NAC-CuNCs) were incorporated into three-dimensional mesoporous silica particles (M-SiO2) through the electrostatic assembly for detecting prostate cancer (PCa) biomarker acid phosphatase (ACP) (Figure 4) [115]. This process enhanced the fluorescence emission and quantum yield of the NAC-CuNCs due to the confinement effect of M-SiO_2_. These nanoprobes were then combined with MnO_2_ nanosheets, a fluorescence quencher, resulting in a fluorescence quenching effect through the inner filter effect. Subsequently, the addition of ACP triggered the hydrolysis of l-ascorbic acid-2-phosphate (AAP) into ascorbic acid (AA). This AA, in turn, facilitated the reduction of MnO_2_ nanosheets into Mn^2+^, thus restoring the fluorescence emission and creating a turn-off/turn-on fluorescent detection platform for ACP. The platform exhibited a detection limit of 0.47 U/L for ACP activity and demonstrated high accuracy in measuring ACP levels in real serum samples.

An all-solid-state (ASS) potentiometric sensor for sarcosine, a biomarker for prostate cancer, has been developed using a molecularly imprinted polymer (MIP) polymerized over silica nanoparticles (Si) [87]. This MIP-Si sensor exhibits high selectivity in phosphate-buffered solution (PBS) and simulated body fluid (SBF). It demonstrates a linear response in the concentration range of 10^−5^–10^−8^ mol/L, with a detection limit of 7.8 × 10^−8^ mol/L and a response time of approximately 30 s. The sensor remains stable for at least 150 days, showcasing its stability, reproducibility, and sensitivity for PCa detection. This work introduced the miniaturized potentiometric ASS sensor for sarcosine recognition and highlighted its low limit of detection, quick response time, and wide linear range. The MIP synthesized on silica nanoparticles enables the development of a selective sensor for sarcosine with analytical applicability in PCa diagnostic applications. One principle to overcome the use of enzyme-based colorimetric systems and apply the nanomaterial technology is an improved strategy for nanozyme catalytic performance in color reaction, where PQQ-decorated MSNs act as a nanocatalyst in the reduction of Fe(III)-ferrozine into Fe(II)-ferrozine by Tris(2-carboxyethyl)phosphine (TCEP) [106]. PQQ-decorated MSNs functionalized with anti-PSA antibody 2 are used as an enhanced catalyst of a colorimetric signal, while magnetic beads functionalized with anti-PSA antibody 1 are used as a capture probe. Using the sandwich-type mechanism, with PSA as a bridge between the capture nanoprobe and nanocatalyst, a colorimetric signal was measured using UV-Vis absorption spectra, and LOD was estimated to be 1 pg/mL. MSNs can play a significant role in fluorescent signal amplification, i.e., the “turn-on” approach, where they are loaded with fluorophores [120]. Particularly, MSNs are functionalized with luminous CdTe quantum dots with two emission wavelengths and adsorbed on the quenching surface of MoS_2_ nanosheets via target-specific aptamers; once aptamers bind target molecules, namely PSA and CEA, MSNs are desorbed, and fluorescence is turned-on. This dual-fluorescence mechanism enabled the ultrasensitive detection of two cancer biomarkers, with LOD of 0.7 fg/mL for CEA and 0.9 fg/mL for PSA.

### 4.4. Cervical Cancer

Cervical cancer ranks as the fourth most common cancer in women, both in terms of new cases and deaths. In 2022, there were approximately 660,000 new diagnoses and 350,000 fatalities globally [17].

MSNs can serve as a redox probe nano-depot, which can be released to amplify the electrochemical signal upon target capture by the bioreceptor. In that sense, MSNs are loaded with MB, capped with chitosan, and additionally functionalized with anti-E6 antibody 2 for the detection of HPV16 E6 oncoprotein [92]. Moreover, a glassy carbon electrode (WE) was modified with innovative dendritic palladium–boron–phosphorus nanospheres (PdBP-NSs) and anti-E6 antibody 1 for specific E6 capture. Owing to the sandwich-type interaction mechanism, the biosensor was able to detect as low as 34.1 fg/mL in a broad dynamic range of 50 fg/mL–4 ng/mL. The use of MSNs reduces the electro-polymerization of MB during the reaction and amplifies the signal response.

A sandwich-type electrochemical immunosensor was developed for ultrasensitive detection of squamous cell carcinoma antigen (SCCA), a common biomarker for cervical cancer [96]. Highly branched PtCo nanocrystals (PtCo BNCs) were synthesized via a solvothermal reaction to serve as electrode substrates, enhancing conductivity and providing active sites for antibody (Ab1) loading. Dendritic mesoporous SiO_2_@AuPt nanoparticles (DM-SiO_2_@AuPt NPs) were prepared through wet chemical methods and used to adsorb thionine (Thi) as a signal label, increasing detection sensitivity. PtCo BNCs facilitated electron transfer and Ab1 loading, while DM-SiO2@AuPt NPs enhanced Thi loading and captured the secondary antibody (Ab2). The combination of PtCo BNCs and DM-SiO2@AuPt NPs amplified electrochemical signals, enabling sensitive SCCA detection. The sensor exhibited a linear range from 0.001 to 120 ng/mL and a detection limit of 0.33 pg/mL with high reproducibility and acceptable recovery in diluted human serum samples.

### 4.5. Pancreatic Cancer

A controlled release of glucose from MSN pores, which is used as an active component in electrochemical reactions on the modified WE, is achieved to successfully detect CA 19-9 [90]. Glucose-loaded MSNs are capped with ZnS, modified with anti-CA19-9 antibody 2 (ZnS@MSN-Glu-Ab2), and act as a signal amplifier when bound to CA19-9 previously captured by antibody 1 in a reaction well. Only CA19-9-anchored MSNs will undergo uncapping via DTT cleaving of disulfide bonds, which releases glucose. Finally, an electrochemical signal was developed using novel 3D cactus-like nickel–cobalt-layered double hydroxide on copper selenide nanosheet-modified carbon cloth (NiCo-LDH/CuSe/CC) with enhanced electrochemical activity for glucose oxidation. Glucose oxidation was monitored using a DPV and a very low concentration of only 0.0005 U/mL was calculated as a limit of detection. Researchers also introduced a novel approach for the ultrasensitive detection of GPC1, a potential biomarker for pancreatic cancer, through a photoelectrochemical (PEC) immunosensor utilizing gold nanoclusters (AuNCs) [101]. Furthermore, the study extended this technique to develop a multichannel light-addressable PEC sensor capable of simultaneously detecting GPC1, carcinoembryonic antigen (CEA), and glutathione (GSH). This sensor combines AuNC/GO-based PEC immunosensors for GPC1 and CEA detection with carbon dots@mesoporous silica bead (CDs@MSB)-based PEC sensors for GSH detection. The combined sensor demonstrates high sensitivity and specificity, achieving accurate and simultaneous detection of the biomarkers in cell, mouse, and patient models of pancreatic cancer. Compared to commercial kits, the light-addressable sensor offers superior sensitivity, lower detection limits, and faster detection times, with robust anti-interference capabilities in complex biological environments. Overall, this innovative sensor holds promise for advancing the diagnosis of pancreatic cancer, and the authors suggest future expansion to incorporate additional biomarkers for enhanced diagnostic accuracy and sensitivity.

### 4.6. Ovarian Cancer

Liu et al. investigated an approach utilizing BDMSNs combined with multiplex lateral flow immunoassay (MLFIA) for the simultaneous detection of ovarian cancer biomarkers CA 125 and HE4 [121]. The BDMSNs serve as fluorescent signal reporters and demonstrate robust antibody enrichment properties due to their aggregation-induced emission property and high affinity for the biotin–streptavidin system. The linear ranges for CA125 and HE4 detection were found to be 0.1–1000 U/mL and 1–1000 pM, respectively, with corresponding limits of detection of 5 U/mL and 5 pM. The coefficient of variation for intra-assay and inter-assay were both less than 15%. Furthermore, the developed BDMSN-MLFIA showed no cross-reactivity with common tumor markers (AFP, CA 199, CEA), and the clinical test results demonstrated a correlation coefficient of over 98% when compared with commercial electrochemiluminescence methods. A sandwich-type magneto-immunosensor was developed for the simultaneous detection and quantification of three ovarian cancer biomarkers: HE4, AFP, and CA 125 [100]. The immunosensor employs bioaffinity interactions of target molecules with specific antibodies and uses screen-printed electrodes combined with electroactive nanomaterials, including gold nanoparticles (AuNPs) and CdTe and PbS QDs. These nanomaterials are conjugated with specific antibodies and integrated with mesoporous silica nanoparticles (SiNPs) for enhanced electrochemical signals.

### 4.7. Other Cancers

Fei et al. constructed and evaluated a GSH-triggered nanoreactor, developed using mesoporous silica nanoparticles (MSNs) coated with a MOF shell formed by coordinating Cu(II) with trimesic acid [116]. The Cu(I) species, generated via GSH-mediated reduction, acts as a catalyst to accelerate azide–alkyne 1,3-cycloaddition (CuAAC) reactions. The nanoreactor demonstrates good biocompatibility and efficacy in GSH sensing, both in cellular environments and in wheat plumules. Specifically, it exhibits high specificity and sensitivity to GSH, with a minimum detection concentration of 0.025 mM in vitro. Additionally, it enables the visualization of GSH distribution within single living cells, unlike traditional electrochemical methods. Moreover, fluorescence signals indicate the influence of Cd^2+^ and Pb^2+^ ions on GSH expression in wheat plumules. The nanoreactor’s unique properties suggest promising applications in intracellular sensing of various analytes, disease diagnostics, and agricultural research.

An electrochemical cytosensor was developed to detect HT-29 colorectal cancer stem cells (CSCs) using a nanocomposite of mesoporous silica nanoparticles (MSNs) and platinum nanoparticles (PtNPs) on a GCE [99]. The PtNPs, approximately 100 nm in size, were electrodeposited onto the MSN substrate, providing high-rate porosity and increased surface-to-volume ratio, facilitating efficient binding of biotinylated monoclonal antibodies targeting CD133, a CSC marker. DPV and SWV confirmed reduced charge transfer and electrical current upon interaction with CD133+ cells. The cytosensor demonstrated sensitivity to detect CSCs ranging from 5 to 20 cells/5 μL, outperforming flow cytometry. The integration of MSNs and PtNPs enhanced mass and charge transfer rates, providing active sites for antibody binding.

Another paper introduced a novel dual-signal-amplified sandwich-type electrochemical immunoassay for the detection of CEA [91]. By utilizing dual-labeled mesoporous silica nanospheres (amine-functionalized SBA-15 entrapping Au nanorod followed by covalent conjugation of HRP and antibody (anti-CEA, Ab2)) as signal amplifiers, combined with NiO@Au- and anti-CEA (Ab1)-decorated graphene as a conductive layer, they achieved remarkable sensitivity enhancement. The synthesized dual-labeled mesoporous silica (DLMS) nanospheres demonstrated ultra-low limits of detection (5.25 fg/mL) and a wide linear range (0.1–5 pg/mL) measured by DPV. The developed immunosensor also showed as an appropriate system in terms of selectivity, detecting no significant impact of different interfering proteins. Furthermore, the DLMS-based immunosensor exhibited excellent performance in real-time CEA determination, with significantly improved recoveries (>98%), confirmed by a typical spiking technique on human serum samples and a commercially accessible method (ELISA). This innovative approach holds promise for meeting the clinical demand for ultrasensitive detection of CEA biomarkers, thereby contributing to early cancer diagnosis and disease progression monitoring.

In a separate study, researchers developed a 3D electrochemical sensing interface for sialic acid (SA) utilizing a mesoporous–macroporous structure created through a layer-by-layer assembly method [94]. The interface was constructed on electrode surfaces using polystyrene (PS) microtubes coated with mesoporous silica and loaded with sambucus nigra agglutinin (SNA). The detection was based on the specific recognition of SNA and SA. The interface demonstrated enhanced cellular capture efficiency and specific recognition of SA overexpressed on cancer cell surfaces. By employing a layer-by-layer assembly method, the exposure of active substances was maximized, resulting in better cellular capture performance compared to direct mixing methods. The 3D structure of the PS nanotubes increased the electrode’s specific surface area, improving its efficiency in capturing cancer cells. Additionally, the mesoporous structure facilitated the loading of more SNA, enhancing the specific recognition of cancer cells. The developed cytosensor exhibited a linear detection range of 1−1.0 × 10^7^ cells/m and a detection limit of 4 cells/mL (S/N = 3).

A controlled-release MSN-based nanoprobe was developed for detecting Flap endonuclease 1 (FEN1), a structure-specific nuclease that catalyzes the removal of a 5′ overhanging DNA flap from a specific DNA structure [117]. They entrapped the fluorescence molecule Rh6G using gold nanoparticles linked to specific single-stranded DNA (AuNPs-ssDNA) as a molecular gate. The presence of FEN1 cleaves the ssDNA, resulting in the release of Rh6G and the recovery of fluorescence. They demonstrated a good linear relationship with the logarithm of FEN1 activity ranging from 0.05 to 1.75 U with a detection limit of 0.03 U. Furthermore, it has been suggested that biosensors could distinguish tumor cells from normal cells. Further, a mesoporous silica-based nanotheranostic system targeting MUC-1-positive tumor cells (MCF-7 and HT-29) was developed [119]. It involves encapsulating curcumin into chitosan–triphosphate nanoparticles, which are then loaded into a nanosystem consisting of mesoporous silica, chitosan, and gold, targeted by an aptamer. The nanosystem enables targeted imaging and drug delivery, with the aptamer triggering drug release upon binding to MUC-1 receptors. The system shows selective toxicity towards MUC-1-positive cells and it is proposed for cancer diagnosis, imaging, and therapy. However, to form the highly sensitive biosensor, optimization of the threshold concentration of the aptamer is needed.

Another paper presents a reverse-phase microemulsion synthesizing method for obtaining silica nanoparticles and incorporating chitosan and the fluorescent dye lucigenin during the reaction [118]. Chitosan addition enhances nanoparticle porosity and facilitates lucigenin molecule integration, increasing fluorescence quantum yield compared to lucigenin/silica NPs without chitosan. Target DNA/miRNA was hybridized with biotin-labeled probe DNA fixed onto the surface of the magnetic beads. Target DNA/miRNA detection relied on the distinct fluorescence responses observed between single-stranded DNA (ssDNA) and double-stranded DNA (dsDNA). The composite nanoparticles exhibit discriminative fluorescence intensity based on the charge difference between single-stranded DNA (ssDNA) and double-stranded DNA (dsDNA), enabling direct detection of let-7a in human gastric cancer cell samples without enzymes, labeling, or immobilization. The method demonstrates a detection limit of 10 fM and selectivity, with lucigenin/chitosan/silica composite nanoparticles serving as efficient DNA hybrid indicators. These composite nanoparticles amplify fluorescence signals through mass transfer nanochannels, resulting in enhanced sensitivity for let-7a detection in tumor cells compared to existing methods. Additionally, by modifying the probe DNA on magnetic beads, the composite nanoprobes can detect other biomolecules. Dendritic-large MSNs are synthesized to improve antibody and horseradish peroxidase (HRP) immobilization used for two-step detection of CEA [122]. Namely, HRP is involved in CL intensity enhancement of luminol only in the presence of CEA, which is achieved by magnetic separation of Fe_3_O_4_@SiO_2_-Ab2 microspheres conjugated to MSN-HRP/Ab1 through the antibody 2-CEA-antibody 1 bridge.

MSNs are employed to amplify the SERS signal for methyltransferase activity determination [128]. Here, MSN pores are loaded by a loading DNA and capped by a specifically designed dsDNA, which can be opened by a trigger DNA produced upon the presence of the target enzyme and nicking endonuclease. The loading and trigger DNAs are released, where trigger DNA can repeat the uncapping cycle (amplification step), and the loading DNA undergoes further SERS signal development. For that, functionalized magnetic beads (MBs) with capture DNA and functionalized AuNPs with reporter DNA having a SERS probe (rhodamine-based) are used. The loading DNA is hybridized to both capture and reporter DNAs, which is then separated, and the Raman spectra are recorded. The 0.02 U/mL detection limit of the target enzyme is reached using the novel principle of this method.

A biosensor for the determination of L-lactic acid (LA) has also been developed [88]. The biosensor uses a flow injection analysis (FIA) system with a lactate oxidase (LOx)-based mini-reactor connected to a silver amalgam screen-printed electrode (AgA-SPE) for detection. The mini-reactor contains mesoporous silica (SBA-15) coated with covalently immobilized LOx, enabling a large enzyme loading of approximately 270 μg. This setup ensures high stability, with 93.8% of the initial signal retained after 350 measurements and 96.9% after 7 months. The detection principle is based on the amperometric monitoring of oxygen consumption due to LA oxidation, measured by the four-electron reduction of oxygen at −900 mV vs. Ag pseudo-reference electrode. This method avoids interference from common oxidizing substances like ascorbic and uric acid. The biosensor was tested for LA quantification in saliva, wine, and dairy products, showing high selectivity, stability, and sensitivity, with a limit of detection of 12.0 μmol/L. The design allows for easy replacement of the mini-reactor or reuse of the electrode, making it versatile and practical for clinical diagnostics and food quality control. In another study, an electrochemical aptasensor for detecting lysozyme (Lys) was developed using a nanocomposite of amino-reduced graphene oxide (Amino-rGO), an ionic liquid (1-Butyl-3-methylimidazolium bromide), and amino-mesosilica nanoparticles (Amino-MSNs) [93]. This nanocomposite, integrated into a screen-printed carbon electrode, offers thermal and chemical stability, conductivity, surface-to-volume ratio, cost efficiency, biocompatibility, and bioelectrocatalytic properties. Anti-lysozyme aptamers (anti-Lys aptamers) were covalently coupled to the nanocomposite using glutaraldehyde as a linker, enhancing the electrochemical signal and sensitivity. The aptasensor’s performance was characterized by CV, DPV, and EIS. The presence of lysozyme increased charge transfer resistance in EIS and decreased DPV peak currents, providing analytical signals for lysozyme detection. Two calibration curves were established, demonstrating LOD of 2.1 and 4.2 fmol/L.

## 5. Perspectives and Outlook

Due to the outstanding properties of MSNs, they have substantial benefits in sensing cancer biomarkers (Table 4).

A novel generation of biosensors employing the use of MSNs is on the rise. Besides the high surface area, which is typical for nanoparticles, MSNs offer several unique attributes that bring substantial benefits for devising biosensors. One of these properties is the possibility of stable (covalent) functionalization of their surface. Hence, different types of functional groups, such as thiol, amine, hydroxyl, halogen, and others are easily grafted on the silica surface in a one-step reaction with different alkoxysilanes, which is typically performed in organic solvents and dry conditions, preferably at elevated temperatures to stimulate the evaporation of as-formed nus products (alcohols). Further modification of the surface is subsequently achieved through different possible covalent coupling reactions, such as click reactions (e.g., thiols with maleimide groups [130] or hydroxyl groups with isocyanates), carbodiimide-catalyzed coupling reactions (between amines and carboxylates [131]), or substitution reactions (e.g., the substitution of halogen with nucleophiles such as amine groups [132]). This feature allows the employment of versatile functionalization strategies for achieving the desired final functionalization on the NP surface. Moreover, each functionalization step can be performed through heterogeneous reactions, and hence the modified NPs can be isolated and washed by simple centrifugation. The same covalent functionalization strategies can be used for attaching the desired NPs to the desired 2D surfaces, thus yielding stable biosensing platforms.

The ordered porosity of MSNs is another unique property that brings substantial incentive to their use in devising biosensors. The pores can be loaded with signaling molecules such as dyes or electroactive species for devising optical or electrochemical biosensors, respectively. More importantly, the release of the loaded cargo molecules can be governed by surface-functionalization and pore-blocking species. Thus, large molecules or nanoparticles have been demonstrated for successful capping of the pore entrances and entrapping cargo molecules [133,134]. Furthermore, the on-desire release of cargo molecules can be achieved by binding the pore-blocking species to the MSNs through stimuli-cleavable linkers. For this purpose, the employed linkers contain functional groups within their structure that can be cleaved upon reaction with specific reagents (such as disulfide groups for cleavage by reduction agents, e.g., glutathione), change in pH (such as hydrazone or acetal linkages for cleavage by acidification), or upon exposure to other incentives such as magnetic field or light irradiation.

The advantage of using the loaded MSNs for triggering the release of signaling molecules lies in the possibility of releasing a substantial amount of the loaded molecules per cleavage event, which could lead to highly sensitive detection. Thus, a substantial amplification factor is expected as one cleavage-triggering agent (analyte) could release an abundance of the pore-loaded signaling molecules. It can be envisioned that such a property would be beneficial for releasing dyes or electroactive species for optical or electrochemical sensing platforms, respectively. The fact that MSNs are not optically active and non-conductive without the loaded signaling molecules is also beneficial for enhancing the signal/noise ratio. Nevertheless, the non-conductive nature of these nanoparticles may limit their applicability in some electrochemical sensors. To address this issue, surface modifications with conductive species (polymer, graphene, or noble metal layers) should be considered. In this case, having the MSNs on the surface of the electrodes would be beneficial for the sensing process by enhancing the surface roughness and hence the sensitivity of the sensor.

The development of biosensors for cancer biomarkers based on MSNs is also promising from the aspect of the known procedures for their affordable large-scale production [135,136,137]. However, the use of expensive recognition elements such as antibodies or aptamers could increase the cost of the final products. Nevertheless, the condition of heterogenous post-modification could allow the reuse of the non-reacted recognition elements after centrifugation of MSNs, which could decrease the final costs of biosensors.

Finally, even though silica is known for its stability, such as its low degradability in neutral or acidic conditions, its hydrolysis and dissolution in the presence of basic molecules could limit its applicability in such environments. Thus, the long-term stability of MSNs could be an issue for reusable or prolonged detection of cancer biomarkers in the weakly alkaline physiological environment of blood (pH 7.4) or in urine (pH up to 8). Hence, the development of standard protocols is still needed for suitable functionalization and passivation of the MSNs to increase their stability in weakly basic conditions.

## 6. Conclusions

In general, the unique characteristics of MSNs warrant their vast potential for the construction of efficient optical and electrochemical sensors, which is yet to be realized in full measure through further research. The porosity of MSNs allows loading of the signaling molecules and their possible release triggering in the presence of desired analytes. The formation of stable covalent bonds on the surface of MSNs and between the MSNs and the sensing substrates offers opportunities for the construction of stable biosensing structures. However, the limiting factor in the case of the electrochemical sensors could be their low conductivity, while the low stability of MSNs in alkaline environments could limit their use for prolonged and reusable sensing in weakly alkaline blood and urine samples. Nevertheless, the exceptional capabilities for covalent functionalization of the MSNs surface could enhance their conductivity as well as their stability in alkaline conditions and hence allow the construction of affordable and efficient POCT sensors in the future.

## Figures and Tables

**Table 1:** Basic cancer biomarkers and associated cancers.

Cancer Biomarker	Cancer Type	Reference
PSA	Prostate	[37]
IgG	Prostate	[38]
PAP, PSA	Prostate	[39]
Peptide fragments	Colorectal	[40]
MMP	Colorectal	[41]
CEA, CA 19-9, CA A24-2	Colorectal and pancreatic	[42]
P53 gene	Colorectal	[43]
CYFRA 21-1	Lung	[44]
CEA, CA 19-9, SCC antigen, NSE	Lung	[45]
EVOM	Breast	[46]
EGFR, HER2, transmembrane glycoproteins CD44 and CD24	Breast	[47]
Sialic acid	Breast and liver	[48]
AFP	Liver	[49]
CA 125, HE4	Ovarian	[50]
TRP-2, NY-ESO-1 melanoma Antigen	Melanoma	[51]

Abbreviations: IgG—immunoglobulin G; PAP—prostatic acid phosphatase; MMP—matrix metalloproteinase; CA—cancer antigen; CYFRA—cytokeratin fragment; SCC—squamous cell carcinoma; EVOM—endogenous volatile organic metabolites; EGFR—epidermal growth factor receptor; HER—human epidermal growth factor receptor; SA—sialic acid; AFR—α-fetoprotein; HE—human epididymis protein; TRP—tyrosinase-related protein.

**Table 2:** An overview of electrochemical techniques used for the biosensing of cancer biomarkers with MSNs applied to enable or enhance the signal of detection.

Technique	Method	MSN Role	Target Biomarker	Key Performances	Reference
Potentiometry	Commercial glucometer	Release of glucose upon target cDNA hybridization	miRNA-21	50 pM–5 nM ^1^ 19 pM ^2^	[84]
		Release of glucose upon target binding to antibody	CYFRA 21-1	1.3–160 ng/mL ^1^	[85]
	Open circuit voltage	Release of [Fe(CN)_6_]^3−^ upon target cDNA hybridization	miRNA-21	10 aM–1 pM ^1^	[86]
	Chrono-potentiometry	MIP performance improvement	Sarcosine	10 nM–10 μM ^1^ 7.8 Nm ^2^	[87]
Amperometry	Chrono-amperometry	Lactate oxidase immobilization	Lactic acid	40–500 μM ^1^	[88]
Voltammetry	Cyclic voltammetry	Antibody immobilization with AgNP for electron transfer improvement	PSA	50 pg/mL–50 ng/mL ^1^ 15 pg/mL ^2^	[89]
	Differential pulse voltammetry	Release of glucose from target-bound MSNs	CA 19-9	0.01–100 U/mL ^1^ 0.0005 U/mL ^2^	[90]
		Dual-labeled MSNs with AuNRs and HRP for signal enhancement	CEA	0.1–5 pg/mL ^1^ 5.25 fg/mL ^2^	[91]
		Sandwich-type immunoassay with MB@MSNs for signal enhancement	HPV16 E6 oncoprotein	50 fg/mL–4 ng/mL ^1^	[92]
		Amino-MSNs in composite with Amino-rGO and IL for signal enhancement	Lysozyme	20 fM–50 nM ^1^	[93]
		SNA-loaded MSNs for improved capture of target	MCF-7 cancer cells	1−1.0 × 107 cells/mL ^1^ 4 cells/mL ^2^	[94]
		Sandwich-type immunoassay with MMSN@AuNP-Ab2 for signal enhancement	CYFRA 21-1	0.01–1.0 pg/mL ^1^ 2 fg/mL ^2^	[95]
		Sandwich-type immunoassay with thionine-loaded MSNs for signal enhancement	SCCA	0.01–120 ng/mL ^1^ 0.33 pg/mL ^2^	[96]
	Square wave voltammetry	Sandwich-type immunoassay with MB-loaded MSNs for signal production by controlled MB release	PSA	10 fg/mL–100 ng/mL ^1^ 1.25 fg/mL ^2^	[97]
		Release of MB from programmed target-enabled CHA for HCR signal amplification	miRNA-21	0.1 fM–5 pM ^1^	[98]
		Sensitivity improvement by MSNs/PtNPs	CD133	5–20 cells/5 μL ^1^	[99]
	Square wave anodic/cathodic stripping voltammetry	Nanocomposites for signal development and enhancement: PbS-QD@MSNs, CdTe-QD@MSNs, and AuNPs@MSNs	HE4, CA-125, and AFP	HE4: 0.02–20 pM ^1^; LOD 5.07 pM CA-125: 0.45–450 IU/L ^1^; LOD 3.1 IU/L AFP: 0.1–500 ng/L ^1^; LOD 2.44 pg/L	[100]
Impedimetry	Electrochemical impedance spectroscopy	Amino-MSNs in composite with Amino-rGO and IL for signal enhancement	Lysozyme	10 fM–200 nM ^1^	[93]
Photoelectrochemical method	Chrono-amperometry	CD@MSB for improved sensitivity	Glutathione	34.9 nM ^2^	[101]

^1^ Linear range. ^2^ LOD. Abbreviations: MIP—molecularly imprinted polymer; cDNA—complementary DNA; Ag NPs—silver nanoparticles; AuNRs—gold nanorods; HRP—horseradish peroxidase; MB—methylene blue; HPV16—human papillomavirus 16; rGO—reduced graphene oxide; IL—ionic liquid; SNA—sambucus nigra agglutinin; MMSN—magnetic MSN; AuNPs—gold nanoparticles; CHA—catalytic hairpin assembly; HCR—hybridization chain reaction; PtNPs—platinum nanoparticles; PbS QDs—lead sulfide quantum dots; CdTe QDs—cadmium telluride quantum dots; and CDs—carbon dots.

**Table 3:** An overview of optical techniques used for the biosensing of cancer biomarkers with MSNs applied to enable or enhance the signal of detection.

Type	Method	MSN Role	Target Biomarker	Key Performances	Reference
Colorimetric	Enzyme based	AuNC-loaded MSNs for improved signal	HER2	10–1000 cells ^1^ 10 cells ^2^	[113]
	Non-enzyme based	DMSN-enabled signal development using CPT/DM-FA nanozyme	GSH	5–80 μM ^1^ 0.654 μM ^2^	[114]
		PQQ-decorated MSNs for sandwich-type signal enhancer	PSA	5–500 pg/mL ^1^ 1 pg/mL ^2^	[106]
Fluorescence	Inner filter effect	CuNC-loaded MSNs for improved fluorescence signal	ACP	0.5–28 U/L ^1^ 0.47 U/L ^2^	[115]
		Nanoreactor based on Cu-MOF-MSNs for signal enhancement	GSH	0–0.1 mM ^1^ 25 μM ^2^	[116]
		Release of Rh6G from MSNs upon ssDNA-AuNP cleaving by target	Flap endonuclease 1	0.05–1.75 U ^1^ 0.03 U ^2^	[117]
		Hybridization-manipulated signal on Luc/CS/MSNs	let-7a (miRNA)	30 fM–9 pM ^1^ 10 fM ^2^	[118]
	Forster resonance energy transfer (FRET)	Aptamer-enabled signal on/off in MSN nanosystem with CS(cur)NPs and AuNPs	MUC-1 (CA 15-3)	-	[119]
		Aptamer-enabled signal development using QD@MSNs	PSA and CEA	PSA: 1 fg/mL–0.1 ng/mL ^1^; 0.9 fg/mL ^2^ CEA: 1 fg/mL–10 pg/mL ^1^; 0.7 fg/mL ^2^	[120]
	Lateral-flow immunoassay	Sandwich-type signal development using BDMSNs	CA 125 and HE4	CA125: 0.1–1000 U/mL ^1^; 5 U/mL ^2^ HE4: 1–1000 pM ^1^; 5 pM ^2^	[121]
Chemiluminescence		Signal amplification by HRP-Ab1@MSNs	CEA	10 pg/mL–20 ng/mL ^1^ 3 pg/mL ^2^	[122]
Electrochemiluminescence		Signal enhancement by CS-Lu-modified SBMMs	SKBR-3	20–2000 cells/mL ^1^ 20 cells/mL ^2^	[37]
		DMSN-enabled signal development using CPT/DM-FA nanozyme	GSH	10–250 μM ^1^ 0.654 μM ^2^	[114]
		Controlled release of Ru(dcbpy)_3_^2+^ from PBA-MSNs	MCF-7	3 × 10^2^–10^5^ cells 208 cells	[123]
		Ru(dcbpy)_3_^2+^-loaded MSNs with dual-quenching signal development	CA 15-3	5.0 × 10^–5^–6.0 × 10^2^ U/mL ^1^ 2.4 × 10^–6^ U/mL ^2^	[124]
		Controlled release of luminol-Ab2 from MSN-PEI upon target binding and pH-stimuli response	CYFRA 21-1	1 fg/mL–100 ng/mL ^1^ 0.4 fg/mL ^2^	[125]
		TPE-TEA-encapsulated MSNs for signal enhancement using DNA strand displacement strategy	MCF-7 cells	10 pg/mL–100 ng/mL ^1^	[126]
Surface plasmon resonance	Plasmonic energy resonance transfer	MSN-enabled Au nanocrescent antenna (MONA)	MCF-7 cancer cells	-	[127]
Other	UV-Vis spectrometry	DMSN-enabled signal development using CPT/DM-FA nanozyme	GSH	2–60 μΜ ^1^ 0.654 μM ^2^	[114]
	Surface-enhanced Raman spectroscopy	Target-enabled signal development by specific DNA release from MSNs	Methyltransferase	0.1–10 U/mL ^1^ 0.02 U/mL ^2^	[128]

^1^ Linear range. ^2^ LOD. Abbreviations: AuNC—gold nanocluster; DMSN—dendritic mesoporous silica nanoparticle; CPT—camptothecin; FA—folic acid; GSH—glutathione; PQQ—pyrroloquinoline quinone; CuNC—copper nanocluster; ACP—acid phosphatase; MOF—metal–organic framework; Rh6G—rhodamine 6G; Luc—lucigenin; CS—chitosan; cur—curcumin; QDs—quantum dots; BDMSNs—biotin-enriched dendritic mesoporous silica nanoparticles; Lu—luminol; Ru(dcbpy)_3_^2+^—Tris(bipyridine)ruthenium(II) chloride; PBA—phenylboronic acid; PEI—polyethylenimine; TPE—tetraphenylethylene; and TEA—triethylamine.

**Table 4:** The impact of the physicochemical properties of MSNs on their performance as detection materials.

Properties	Benefits	Challenges	Applications Related to Sensing Cancer Biomarkers
High surface area	Surface functionalization with different molecules.	Controlling the amount and distribution of surface functional groups.	High amount of receptors for interaction with analytes or for attachment to sensing surfaces for optical or electrochemical detection with low LOD.
Porosity	Uniform distribution of pores with small diameter (2–3 nm), which can be used to load and entrap cargo molecules.	Optimization of porous structure to enhance the capacity for storing and entrapping molecules.	Loading signaling molecules (analytes) and their controlled release for optical or electrochemical sensing.
High stability	Facile formation of stable covalent linkages in reaction with organosilanes. Stability in testing media.	Achieving enhanced degradation for in vivo applications. Long-term stability in weakly alkaline media can present a challenge to achieving sensors for prolonged operation.	Formation of stable sensing surfaces for possible reusable detection.
Biocompatibility	Due to its biocompatibility, the use of silica is approved for cosmetics use.	Achieving approvement for in vivo diagnostics.	Possible construction of wearable biosensors.
Low costs	Highly scalable synthesis with cheap reactants and does not require high purity of chemicals.	The need for the use of expensive recognition elements in post-synthesis modification for specific and selective sensing.	Possible application for affordable POCT detection.

## Data Availability

Not applicable.

## References

[1] Wu S.-H., Mou C.-Y., Lin H.-P. (2013). Synthesis of Mesoporous Silica Nanoparticles. Chem. Soc. Rev..

[2] Narayan R., Nayak U.Y., Raichur A.M., Garg S. (2018). Mesoporous Silica Nanoparticles: A Comprehensive Review on Synthesis and Recent Advances. Pharmaceutics.

[3] Nooney R.I., Thirunavukkarasu D., Chen Y., Josephs R., Ostafin A.E. (2002). Synthesis of Nanoscale Mesoporous Silica Spheres with Controlled Particle Size. Chem. Mater..

[4] Kankala R.K., Han Y., Na J., Lee C., Sun Z., Wang S., Kimura T., Ok Y.S., Yamauchi Y., Chen A. (2020). Nanoarchitectured Structure and Surface Biofunctionality of Mesoporous Silica Nanoparticles. Adv. Mater..

[5] Sancenón F., Pascual L., Oroval M., Aznar E., Martínez-Máñez R. (2015). Gated Silica Mesoporous Materials in Sensing Applications. ChemistryOpen.

[6] Manzano M., Vallet-Regí M. (2018). Mesoporous Silica Nanoparticles in Nanomedicine Applications. J. Mater. Sci. Mater. Med..

[7] Rastogi A., Tripathi D.K., Yadav S., Chauhan D.K., Živčák M., Ghorbanpour M., El-Sheery N.I., Brestic M. (2019). Application of Silicon Nanoparticles in Agriculture. 3 Biotech.

[8] Zamboulis A., Moitra N., Moreau J.J.E., Cattoën X., Wong Chi Man M. (2010). Hybrid Materials: Versatile Matrices for Supporting Homogeneous Catalysts. J. Mater. Chem..

[9] Aguilar Z.P. (2013). Nanobiosensors. Nanomaterials for Medical Applications.

[10] Bhalla N., Jolly P., Formisano N., Estrela P. (2016). Introduction to Biosensors. Essays Biochem..

[11] Naresh V., Lee N. (2021). A Review on Biosensors and Recent Development of Nanostructured Materials-Enabled Biosensors. Sensors.

[12] Tothill I.E. (2009). Biosensors for Cancer Markers Diagnosis. Semin. Cell Dev. Biol..

[13] Singhal J., Verma S., Kumar S., Mehrotra D. (2021). Recent Advances in Nano-Bio-Sensing Fabrication Technology for the Detection of Oral Cancer. Mol. Biotechnol..

[14] Jafari S., Derakhshankhah H., Alaei L., Fattahi A., Varnamkhasti B.S., Saboury A.A. (2019). Mesoporous Silica Nanoparticles for Therapeutic/Diagnostic Applications. Biomed. Pharmacother..

[15] Kholafazad Kordasht H., Pazhuhi M., Pashazadeh-Panahi P., Hasanzadeh M., Shadjou N. (2020). Multifunctional Aptasensors Based on Mesoporous Silica Nanoparticles as an Efficient Platform for Bioanalytical Applications: Recent Advances. TrAC Trends Anal. Chem..

[16] Qasim Almajidi Y., Althomali R.H., Gandla K., Uinarni H., Sharma N., Hussien B.M., Alhassan M.S., Mireya Romero-Parra R., Singh Bisht Y. (2023). Multifunctional Immunosensors Based on Mesoporous Silica Nanomaterials as Efficient Sensing Platforms in Biomedical and Food Safety Analysis: A Review of Current Status and Emerging Applications. Microchem. J..

[17] Bray F., Laversanne M., Sung H., Ferlay J., Siegel R.L., Soerjomataram I., Jemal A. (2024). Global Cancer Statistics 2022: GLOBOCAN Estimates of Incidence and Mortality Worldwide for 36 Cancers in 185 Countries. CA Cancer J. Clin..

[18] Tabassum D.P., Polyak K. (2015). Tumorigenesis: It Takes a Village. Nat. Rev. Cancer.

[19] Wodarz A., Näthke I. (2007). Cell Polarity in Development and Cancer. Nat. Cell Biol..

[20] Soper S.A., Brown K., Ellington A., Frazier B., Garcia-Manero G., Gau V., Gutman S.I., Hayes D.F., Korte B., Landers J.L. (2006). Point-of-Care Biosensor Systems for Cancer Diagnostics/Prognostics. Biosens. Bioelectron..

[21] Henry N.L., Hayes D.F. (2012). Cancer Biomarkers. Mol. Oncol..

[22] Barhoum A., Altintas Z., Devi K.S.S., Forster R.J. (2023). Electrochemiluminescence Biosensors for Detection of Cancer Biomarkers in Biofluids: Principles, Opportunities, and Challenges. Nano Today.

[23] Wagner P.D., Verma M., Srivastava S. (2004). Challenges for Biomarkers in Cancer Detection. Ann. N. Y. Acad. Sci..

[24] Jayanthi V.S.P.K.S.A., Das A.B., Saxena U. (2017). Recent Advances in Biosensor Development for the Detection of Cancer Biomarkers. Biosens. Bioelectron..

[25] Wu L., Qu X. (2015). Cancer Biomarker Detection: Recent Achievements and Challenges. Chem. Soc. Rev..

[26] Gan S.D., Patel K.R. (2013). Enzyme Immunoassay and Enzyme-Linked Immunosorbent Assay. J. Investig. Dermatol..

[27] Kubista M., Andrade J.M., Bengtsson M., Forootan A., Jonák J., Lind K., Sindelka R., Sjöback R., Sjögreen B., Strömbom L. (2006). The Real-Time Polymerase Chain Reaction. Mol. Asp. Med..

[28] Land K.J., Boeras D.I., Chen X.-S., Ramsay A.R., Peeling R.W. (2018). REASSURED Diagnostics to Inform Disease Control Strategies, Strengthen Health Systems and Improve Patient Outcomes. Nat. Microbiol..

[29] Khan H., Shah M.R., Barek J., Malik M.I. (2023). Cancer Biomarkers and Their Biosensors: A Comprehensive Review. TrAC Trends Anal. Chem..

[30] Füzéry A.K., Levin J., Chan M.M., Chan D.W. (2013). Translation of Proteomic Biomarkers into FDA Approved Cancer Diagnostics: Issues and Challenges. Clin. Proteom..

[31] Wu J., Fu Z., Yan F., Ju H. (2007). Biomedical and Clinical Applications of Immunoassays and Immunosensors for Tumor Markers. TrAC Trends Anal. Chem..

[32] Sanchez-Carbayo M. (2004). Recent Advances in Bladder Cancer Diagnostics. Clin. Biochem..

[33] Gann P.H. (1995). A Prospective Evaluation of Plasma Prostate-Specific Antigen for Detection of Prostatic Cancer. JAMA.

[34] Gold P., Freedman S.O. (1965). Demonstration of Tumor-Specific Antigens in Human Colonic Carcinomata by Immunological Tolerance and Absorption Techniques. J. Exp. Med..

[35] Mordente A., Meucci E., Martorana G.E., Silvestrini A., Scatena R. (2015). Cancer Biomarkers Discovery and Validation: State of the Art, Problems and Future Perspectives. Advances in Cancer Biomarkers.

[36] Ross J.S., Fletcher J.A., Bloom K.J., Linette G.P., Stec J., Symmans W.F., Pusztai L., Hortobagyi G.N. (2004). Targeted Therapy in Breast Cancer. Mol. Cell. Proteom..

[37] Nasrollahpour H., Mahdipour M., Isildak I., Rashidi M.-R., Naseri A., Khalilzadeh B. (2021). A Highly Sensitive Electrochemiluminescence Cytosensor for Detection of SKBR-3 Cells as Metastatic Breast Cancer Cell Line: A Constructive Phase in Early and Precise Diagnosis. Biosens. Bioelectron..

[38] Zheng T., Pierre-Pierre N., Yan X., Huo Q., Almodovar A.J.O., Valerio F., Rivera-Ramirez I., Griffith E., Decker D.D., Chen S. (2015). Gold Nanoparticle-Enabled Blood Test for Early Stage Cancer Detection and Risk Assessment. ACS Appl. Mater. Interfaces.

[39] Epstein J.I. (1993). PSA and PAP as Immunohistochemical Markers IN Prostate Cancer. Urol. Clin. N. Am..

[40] Li X., Tan J., Yu J., Feng J., Pan A., Zheng S., Wu J. (2014). Use of a Porous Silicon–Gold Plasmonic Nanostructure to Enhance Serum Peptide Signals in MALDI-TOF Analysis. Anal. Chim. Acta.

[41] Schuerle S., Dudani J.S., Christiansen M.G., Anikeeva P., Bhatia S.N. (2016). Magnetically Actuated Protease Sensors for in Vivo Tumor Profiling. Nano Lett..

[42] Rao H., Wu H., Huang Q., Yu Z., Zhang Q., Zhong Z. (2021). Clinical Diagnostic Value for Colorectal Cancer Based on Serum CEA, CA24-2 and CA19-9. Clin. Lab..

[43] Tokunaga R., Sakamoto Y., Nakagawa S., Yoshida N., Baba H. (2017). The Utility of Tumor Marker Combination, Including Serum P53 Antibody, in Colorectal Cancer Treatment. Surg. Today.

[44] Lu N., Gao A., Dai P., Mao H., Zuo X., Fan C., Wang Y., Li T. (2015). Ultrasensitive Detection of Dual Cancer Biomarkers with Integrated CMOS-Compatible Nanowire Arrays. Anal. Chem..

[45] Yang Y., Chang S., Wang N., Song P., Wei H., Liu J. (2023). Clinical Utility of Six Serum Tumor Markers for the Diagnosis of Lung Cancer. iLABMED.

[46] Qiao Z., Perestrelo R., Reyes-Gallardo E.M., Lucena R., Cárdenas S., Rodrigues J., Câmara J.S. (2015). Octadecyl Functionalized Core–Shell Magnetic Silica Nanoparticle as a Powerful Nanocomposite Sorbent to Extract Urinary Volatile Organic Metabolites. J. Chromatogr. A.

[47] Wang Y.W., Doerksen J.D., Kang S., Walsh D., Yang Q., Hong D., Liu J.T.C. (2016). Multiplexed Molecular Imaging of Fresh Tissue Surfaces Enabled by Convection-Enhanced Topical Staining with SERS-Coded Nanoparticles. Small.

[48] Zhang X., Chen B., He M., Zhang Y., Peng L., Hu B. (2016). Boronic Acid Recognition Based-Gold Nanoparticle-Labeling Strategy for the Assay of Sialic Acid Expression on Cancer Cell Surface by Inductively Coupled Plasma Mass Spectrometry. Analyst.

[49] Zhao Y.-J., Ju Q., Li G.-C. (2013). Tumor Markers for Hepatocellular Carcinoma. Mol. Clin. Oncol..

[50] Rao S., Smith D.A., Guler E., Kikano E.G., Rajdev M.A., Yoest J.M., Ramaiya N.H., Tirumani S.H. (2021). Past, Present, and Future of Serum Tumor Markers in Management of Ovarian Cancer: A Guide for the Radiologist. RadioGraphics.

[51] Khong H.T., Rosenberg S.A. (2002). Pre-Existing Immunity to Tyrosinase-Related Protein (TRP)-2, a New TRP-2 Isoform, and the NY-ESO-1 Melanoma Antigen in a Patient with a Dramatic Response to Immunotherapy. J. Immunol..

[52] Manne U., Srivastava R.-G., Srivastava S. (2005). Keynote Review: Recent Advances in Biomarkers for Cancer Diagnosis and Treatment. Drug Discov. Today.

[53] Balendiran G.K., Dabur R., Fraser D. (2004). The Role of Glutathione in Cancer. Cell Biochem. Funct..

[54] Morris P.E., Bernard G.R. (1994). Significance of Glutathione in Lung Disease and Implications for Therapy. Am. J. Med. Sci..

[55] Kennedy L., Sandhu J.K., Harper M.-E., Cuperlovic-Culf M. (2020). Role of Glutathione in Cancer: From Mechanisms to Therapies. Biomolecules.

[56] Gamcsik M.P., Kasibhatla M.S., Teeter S.D., Colvin O.M. (2012). Glutathione Levels in Human Tumors. Biomarkers.

[57] Moll R., Seifer G. (1987). Epithelial Tumor Markers: Cytokeratins and Tissue Polypeptide Antigen (TPA). Morphological Tumor Markers.

[58] Sundström B.E., Stigbrand T.I. (1994). Cytokeratins and Tissue Polypeptide Antigen. Int. J. Biol. Markers.

[59] Wieskopf B., Demangeat C., Purohit A., Stenger R., Gries P., Kreisman H., Quoix E. (1995). Cyfra 21-1 as a Biologic Marker of Non-Small Cell Lung Cancer. Chest.

[60] Duffy M.J., Shering S., Sherry F., McDermott E., O’Higgins N. (2000). CA 15–3: A Prognostic Marker in Breast Cancer. Int. J. Biol. Markers.

[61] Mitri Z., Constantine T., O’Regan R. (2012). The HER2 Receptor in Breast Cancer: Pathophysiology, Clinical Use, and New Advances in Therapy. Chemother. Res. Pract..

[62] Maetzel D., Denzel S., Mack B., Canis M., Went P., Benk M., Kieu C., Papior P., Baeuerle P.A., Munz M. (2009). Nuclear Signalling by Tumour-Associated Antigen EpCAM. Nat. Cell Biol..

[63] Went P.T.H., Lugli A., Meier S., Bundi M., Mirlacher M., Sauter G., Dirnhofer S. (2004). Frequent EpCam Protein Expression in Human Carcinomas. Hum. Pathol..

[64] Gutman A.B., Gutman E.B. (1938). An “Acid” Phosphatase Occurring in The Serum Of Patients with Metastasizing Carcinoma of the Prostate Gland. J. Clin. Investig..

[65] Henneberry M.O., Engel G., Grayhack J.T. (1979). Acid Phosphatase. Urol. Clin. N. Am..

[66] Singh J., Pasi D.K., Bala M., Kumar A., Singh A., Jakhar R., Sharma A., Saini A. (2023). Evaluation of prostate-specific antigen and total serum acid phosphatase in prostatic carcinoma. Natl. J. Physiol. Pharm. Pharmacol..

[67] Lilja H. (1985). A Kallikrein-like Serine Protease in Prostatic Fluid Cleaves the Predominant Seminal Vesicle Protein. J. Clin. Investig..

[68] Gjertson C.K., Albertsen P.C. (2011). Use and Assessment of PSA in Prostate Cancer. Med. Clin. N. Am..

[69] Burd E.M. (2003). Human Papillomavirus and Cervical Cancer. Clin. Microbiol. Rev..

[70] Chang Y.-F., Yan G.-J., Liu G.-C., Hong Y., Chen H.-L., Jiang S., Zhong Y., Xiyang Y.-B., Hu T. (2021). HPV16 E6 Promotes the Progression of HPV Infection-Associated Cervical Cancer by Upregulating Glucose-6-Phosphate Dehydrogenase Expression. Front. Oncol..

[71] Ghittoni R., Accardi R., Hasan U., Gheit T., Sylla B., Tommasino M. (2010). The Biological Properties of E6 and E7 Oncoproteins from Human Papillomaviruses. Virus Genes.

[72] Kato H., Torigoe T. (1977). Radioimmunoassay for Tumor Antigen of Human Cervical Squamous Cell Carcinoma. Cancer.

[73] Ohara K. (2002). Assessment of Cervical Cancer Radioresponse by Serum Squamous Cell Carcinoma Antigen and Magnetic Resonance Imaging. Obstet. Gynecol..

[74] Hasan S., Jacob R., Manne U., Paluri R. (2019). Advances in Pancreatic Cancer Biomarkers. Oncol. Rev..

[75] Duan L., Hu X., Feng D., Lei S., Hu G. (2013). GPC-1 May Serve as a Predictor of Perineural Invasion and a Prognosticator of Survival in Pancreatic Cancer. Asian J. Surg..

[76] Atallah G.A., Abd Aziz N.H., Teik C.K., Shafiee M.N., Kampan N.C. (2021). New Predictive Biomarkers for Ovarian Cancer. Diagnostics.

[77] Cesewski E., Johnson B.N. (2020). Electrochemical Biosensors for Pathogen Detection. Biosens. Bioelectron..

[78] Tanwar A., Gandhi H.A., Kushwaha D., Bhattacharya J. (2022). A Review on Microelectrode Array Fabrication Techniques and Their Applications. Mater. Today Chem..

[79] Grieshaber D., MacKenzie R., Vörös J., Reimhult E. (2008). Electrochemical Biosensors—Sensor Principles and Architectures. Sensors.

[80] Chaubey A., Malhotra B.D. (2002). Mediated Biosensors. Biosens. Bioelectron..

[81] Cho I.H., Kim D.H., Park S. (2020). Electrochemical biosensors: Perspective on functional nanomaterials for on-site analysis. Biomater. Res..

[82] Dong T., Matos Pires N.M., Yang Z., Jiang Z. (2023). Advances in Electrochemical Biosensors Based on Nanomaterials for Protein Biomarker Detection in Saliva. Adv. Sci..

[83] Huang X., Zhu Y., Kianfar E. (2021). Nano Biosensors: Properties, Applications and Electrochemical Techniques. J. Mater. Res. Technol..

[84] Deng K., Zhang Y., Tong X. (2018). Sensitive Electrochemical Detection of microRNA-21 Based on Propylamine-Functionalized Mesoporous Silica with Glucometer Readout. Anal. Bioanal. Chem..

[85] Lv F., Wang M., Ma H., Hu L., Wei Q., Wu D. (2022). Sensitive Detection of CYFRA21-1 by a Controlled Release Sensor Based on Personal Glucose Meter. Sens. Actuators B Chem..

[86] Gai P., Gu C., Hou T., Li F. (2018). Integration of Biofuel Cell-Based Self-Powered Biosensing and Homogeneous Electrochemical Strategy for Ultrasensitive and Easy-To-Use Bioassays of MicroRNA. ACS Appl. Mater. Interfaces.

[87] Fernández-Puig S., Lazo-Fraga A.R., Korgel B.A., Oza G., Dutt A., Vallejo-Becerra V., Valdés-González A.C., Chávez-Ramírez A.U. (2022). Molecularly Imprinted Polymer-Silica Nanocomposite Based Potentiometric Sensor for Early Prostate Cancer Detection. Mater. Lett..

[88] Tvorynska S., Barek J., Josypcuk B. (2023). High-Performance Amperometric Biosensor for Flow Injection Analysis Consisting of a Replaceable Lactate Oxidase-Based Mini-Reactor and a Silver Amalgam Screen-Printed Electrode. Electrochim. Acta.

[89] Wang H., Zhang Y., Yu H., Wu D., Ma H., Li H., Du B., Wei Q. (2013). Label-Free Electrochemical Immunosensor for Prostate-Specific Antigen Based on Silver Hybridized Mesoporous Silica Nanoparticles. Anal. Biochem..

[90] Li B., Li Y., Li C., Yang J., Liu D., Wang H., Xu R., Zhang Y., Wei Q. (2023). An Ultrasensitive Split-Type Electrochemical Immunosensor Based on Controlled-Release Strategy for Detection of CA19-9. Biosens. Bioelectron..

[91] Krishnan S., He X., Zhao F., Zhang Y., Liu S., Xing R. (2020). Dual Labeled Mesoporous Silica Nanospheres Based Electrochemical Immunosensor for Ultrasensitive Detection of Carcinoembryonic Antigen. Anal. Chim. Acta.

[92] Wen T., Xia C., Yu Q., Yu Y., Li S., Zhou C., Sun K., Yue S. (2022). A Dual-Signal Electrochemical Immunosensor for the Detection of HPV16 E6 Oncoprotein Based on PdBP Dendritic Ternary Nanospheres and MBSi-Chi Nanocomposites. Analyst.

[93] Jamei H.R., Rezaei B., Ensafi A.A. (2019). An Ultrasensitive Electrochemical Anti-Lysozyme Aptasensor with Biorecognition Surface Based on Aptamer/Amino-rGO/Ionic Liquid/Amino-Mesosilica Nanoparticles. Colloids Surf. B Biointerfaces.

[94] Chen H., Wang Z., Liu Z., Niu Q., Wang X., Miao Z., Zhang H., Wei J., Wan M., Mao C. (2021). Construction of 3D Electrochemical Cytosensor by Layer-by-Layer Assembly for Ultra-Sensitive Detection of Cancer Cells. Sens. Actuators B Chem..

[95] Yola M.L., Atar N., Özcan N. (2021). A Novel Electrochemical Lung Cancer Biomarker Cytokeratin 19 Fragment Antigen 21-1 Immunosensor Based on Si_3_N_4_/MoS_2_ Incorporated MWCNTs and Core–Shell Type Magnetic Nanoparticles. Nanoscale.

[96] Zhang J.-X., Lv C.-L., Tang C., Jiang L.-Y., Wang A.-J., Feng J.-J. (2022). Ultrasensitive Sandwich-Typed Electrochemical Immunoassay for Detection of Squamous Cell Carcinoma Antigen Based on Highly Branched PtCo Nanocrystals and Dendritic Mesoporous SiO2@AuPt Nanoparticles. Microchim. Acta.

[97] Zhang D., Li W., Ma Z. (2018). Improved Sandwich-Format Electrochemical Immunosensor Based on “Smart” SiO_2_@polydopamine Nanocarrier. Biosens. Bioelectron..

[98] Cheng H., Li W., Duan S., Peng J., Liu J., Ma W., Wang H., He X., Wang K. (2019). Mesoporous Silica Containers and Programmed Catalytic Hairpin Assembly/Hybridization Chain Reaction Based Electrochemical Sensing Platform for MicroRNA Ultrasensitive Detection with Low Background. Anal. Chem..

[99] Sadi S., Khalilzadeh B., Mahdipour M., Sokouti Nasimi F., Isildak I., Davaran S., Rashidi M.-R., Bani F. (2023). Early Stage Evaluation of Cancer Stem Cells Using Platinum Nanoparticles/CD133+ Enhanced Nanobiocomposite. Cancer Nano.

[100] Kovarova A., Kastrati G., Pekarkova J., Metelka R., Drbohlavova J., Bilkova Z., Selesovska R., Korecka L. (2023). Biosensor with Electrochemically Active Nanocomposites for Signal Amplification and Simultaneous Detection of Three Ovarian Cancer Biomarkers. Electrochim. Acta.

[101] Xiao G., Ge H., Yang Q., Zhang Z., Cheng L., Cao S., Ji J., Zhang J., Yue Z. (2022). Light-Addressable Photoelectrochemical Sensors for Multichannel Detections of GPC1, CEA and GSH and Its Applications in Early Diagnosis of Pancreatic Cancer. Sens. Actuators B Chem..

[102] Rasheed S., Kanwal T., Ahmad N., Fatima B., Najam-ul-Haq M., Hussain D. (2024). Advances and Challenges in Portable Optical Biosensors for Onsite Detection and Point-of-Care Diagnostics. TrAC Trends Anal. Chem..

[103] Zhao X., Dai X., Zhao S., Cui X., Gong T., Song Z., Meng H., Zhang X., Yu B. (2021). Aptamer-Based Fluorescent Sensors for the Detection of Cancer Biomarkers. Spectrochim. Acta Part A Mol. Biomol. Spectrosc..

[104] Wang H., Wu T., Li M., Tao Y. (2021). Recent Advances in Nanomaterials for Colorimetric Cancer Detection. J. Mater. Chem. B.

[105] Khansili N., Rattu G., Krishna P.M. (2018). Label-Free Optical Biosensors for Food and Biological Sensor Applications. Sens. Actuators B Chem..

[106] Xia N., Deng D., Mu X., Liu A., Xie J., Zhou D., Yang P., Xing Y., Liu L. (2020). Colorimetric Immunoassays Based on Pyrroloquinoline Quinone-Catalyzed Generation of Fe(II)-Ferrozine with Tris(2-Carboxyethyl)Phosphine as the Reducing Reagent. Sens. Actuators B Chem..

[107] Son M.H., Park S.W., Sagong H.Y., Jung Y.K. (2023). Recent Advances in Electrochemical and Optical Biosensors for Cancer Biomarker Detection. BioChip J..

[108] Chen S., Yu Y.-L., Wang J.-H. (2018). Inner Filter Effect-Based Fluorescent Sensing Systems: A Review. Anal. Chim. Acta.

[109] Qi H., Zhang C. (2020). Electrogenerated Chemiluminescence Biosensing. Anal. Chem..

[110] Kaur B., Kumar S., Kaushik B.K. (2022). Recent Advancements in Optical Biosensors for Cancer Detection. Biosens. Bioelectron..

[111] Piliarik M., Vaisocherová H., Homola J., Rasooly A., Herold K.E. (2009). Surface Plasmon Resonance Biosensing. Biosensors and Biodetection.

[112] Lin C., Li Y., Peng Y., Zhao S., Xu M., Zhang L., Huang Z., Shi J., Yang Y. (2023). Recent Development of Surface-Enhanced Raman Scattering for Biosensing. J. Nanobiotechnol..

[113] Li M., Lao Y.-H., Mintz R.L., Chen Z., Shao D., Hu H., Wang H.-X., Tao Y., Leong K.W. (2019). A Multifunctional Mesoporous Silica–Gold Nanocluster Hybrid Platform for Selective Breast Cancer Cell Detection Using a Catalytic Amplification-Based Colorimetric Assay. Nanoscale.

[114] Zhang Y., Meng W., Li X., Wang D., Shuang S., Dong C. (2022). Dendritic Mesoporous Silica Nanoparticle-Tuned High-Affinity MnO_2_ Nanozyme for Multisignal GSH Sensing and Target Cancer Cell Detection. ACS Sustain. Chem. Eng..

[115] Chen S., Li Z., Xue R., Huang Z., Jia Q. (2022). Confining Copper Nanoclusters in Three Dimensional Mesoporous Silica Particles: Fabrication of an Enhanced Emission Platform for “Turn off-on” Detection of Acid Phosphatase Activity. Anal. Chim. Acta.

[116] Zeng F., Pan Y., Luan X., Gao Y., Yang J., Wang Y., Song Y. (2021). Copper Metal-Organic Framework Incorporated Mesoporous Silica as a Bioorthogonal Biosensor for Detection of Glutathione. Sens. Actuators B Chem..

[117] Bagheri Hashkavayi A., Cha B.S., Hwang S.H., Kim J., Park K.S. (2021). Highly Sensitive Electrochemical Detection of Circulating EpCAM-Positive Tumor Cells Using a Dual Signal Amplification Strategy. Sens. Actuators B Chem..

[118] Chang Z., Feng J., Zheng X. (2020). A Highly Sensitive Fluorescence Sensor Based on Lucigenin/Chitosan/SiO _2_ Composite Nanoparticles for microRNA Detection Using Magnetic Separation. Luminescence.

[119] Esmaeili Y., Khavani M., Bigham A., Sanati A., Bidram E., Shariati L., Zarrabi A., Jolfaie N.A., Rafienia M. (2022). Mesoporous Silica@chitosan@gold Nanoparticles as “on/off” Optical Biosensor and pH-Sensitive Theranostic Platform against Cancer. Int. J. Biol. Macromol..

[120] Sun Y., Fan J., Cui L., Ke W., Zheng F., Zhao Y. (2019). Fluorometric Nanoprobes for Simultaneous Aptamer-Based Detection of Carcinoembryonic Antigen and Prostate Specific Antigen. Microchim. Acta.

[121] Liu H., Cao J., Ding S.-N. (2022). Simultaneous Detection of Two Ovarian Cancer Biomarkers in Human Serums with Biotin-Enriched Dendritic Mesoporous Silica Nanoparticles-Labeled Multiplex Lateral Flow Immunoassay. Sens. Actuators B Chem..

[122] Liu S., Li J., Zou Y., Jiang Y., Wu L., Deng Y. (2023). Construction of Magnetic Core–Large Mesoporous Satellite Immunosensor for Long-Lasting Chemiluminescence and Highly Sensitive Tumor Marker Determination. Small.

[123] Chen Y., Chen Z., Fang L., Weng A., Luo F., Guo L., Qiu B., Lin Z. (2020). Electrochemiluminescence Sensor for Cancer Cell Detection Based on H2O2-Triggered Stimulus Response System. J. Anal. Test..

[124] Wang W., Feng D., Wang Y., Kan X. (2023). Ruthenium Poly(Ethylenimine)/Gold Nanoparticles Immobilized on Dendritic Mesoporous Silica Nanoparticles for a CA15-3 Electrochemiluminescence Immunosensor via Cu _2_ O@PDA Dual Quenching. ACS Appl. Nano Mater..

[125] Huang Z., Zhao L., Li Y., Wang H., Ma H., Wei Q., Wu D. (2024). Glucose Oxidation Induced pH Stimuli Response Controlled Release Electrochemiluminescence Biosensor for Ultrasensitive Detection of CYFRA 21-1. Talanta.

[126] Jia Y., Du Y., Ru Z., Fan D., Yang L., Ren X., Wei Q. (2023). Aggregation-Induced Electrochemiluminescence Frame of Silica-Confined Tetraphenylethylene Derivative Matrixes for CD44 Detection via Peptide Recognition. Anal. Chem..

[127] Park Y., Yoon H.J., Lee S.E., Lee L.P. (2022). Multifunctional Cellular Targeting, Molecular Delivery, and Imaging by Integrated Mesoporous-Silica with Optical Nanocrescent Antenna: MONA. ACS Nano.

[128] Wang X., Cui M., Zhou H., Zhang S. (2015). DNA-Hybrid-Gated Functional Mesoporous Silica for Sensitive DNA Methyltransferase SERS Detection. Chem. Commun..

[129] Zhang Y., Vaccarella S., Morgan E., Li M., Etxeberria J., Chokunonga E., Manraj S.S., Kamate B., Omonisi A., Bray F. (2023). Global Variations in Lung Cancer Incidence by Histological Subtype in 2020: A Population-Based Study. Lancet Oncol..

[130] Mundžić M., Lazović J., Mladenović M., Pavlović A., Ultimo A., Gobbo O.L., Ruiz-Hernandez E., Santos-Martinez M.J., Knežević N.Ž. (2024). MRI-Based Sensing of pH-Responsive Content Release from Mesoporous Silica Nanoparticles. J. Sol-Gel Sci. Technol..

[131] Mladenović M., Morgan I., Ilić N., Saoud M., Pergal M.V., Kaluđerović G.N., Knežević N.Ž. (2021). pH-Responsive Release of Ruthenium Metallotherapeutics from Mesoporous Silica-Based Nanocarriers. Pharmaceutics.

[132] Miyajima T., Abry S., Zhou W., Albela B., Bonneviot L., Oumi Y., Sano T., Yoshitake H. (2007). Estimation of Spacing between 3-Bromopropyl Functions Grafted on Mesoporous Silica Surfaces by a Substitution Reaction Using Diamine Probe Molecules. J. Mater. Chem..

[133] Yi S., Zheng J., Lv P., Zhang D., Zheng X., Zhang Y., Liao R. (2018). Controlled Drug Release from Cyclodextrin-Gated Mesoporous Silica Nanoparticles Based on Switchable Host–Guest Interactions. Bioconjugate Chem..

[134] Cheng C.-A., Deng T., Lin F.-C., Cai Y., Zink J.I. (2019). Supramolecular Nanomachines as Stimuli-Responsive Gatekeepers on Mesoporous Silica Nanoparticles for Antibiotic and Cancer Drug Delivery. Theranostics.

[135] Kim C., Yoon S., Lee J.H. (2019). Facile large-scale synthesis of mesoporous silica nanoparticles at room temperature in a monophasic system with fine size control. Microporous Mesoporous Mater..

[136] Zhang K., Xu L.-L., Jiang J.-G., Calin N., Lam K.-F., Zhang S.-J., Wu H.-H., Wu G.-D., Albela B., Bonneviot L. (2013). Facile Large-Scale Synthesis of Monodisperse Mesoporous Silica Nanospheres with Tunable Pore Structure. J. Am. Chem. Soc..

[137] Jundale R.B., Sonawane J.R., Palghadmal A.V., Jaiswal H.K., Deore H.S., Kulkarni A.A. (2024). Scaling-up continuous production of mesoporous silica particles at kg scale: Design & operational strategies. React. Chem. Eng..

